# Medicinal plants used by traditional healers in Kancheepuram District of Tamil Nadu, India

**DOI:** 10.1186/1746-4269-2-43

**Published:** 2006-10-07

**Authors:** Chellaiah Muthu, Muniappan Ayyanar, Nagappan Raja, Savarimuthu Ignacimuthu

**Affiliations:** 1 Entomology Research Institute, Loyola College, Chennai – 600 034, India; 2 Department of Applied Biology, University of Gondar, Gondar, Ethiopia

## Abstract

An ethnobotanical survey was undertaken to collect information from traditional healers on the use of medicinal plants in Kancheepuram district of Tamil Nadu during October 2003 to April 2004. The indigenous knowledge of local traditional healers and the native plants used for medicinal purposes were collected through questionnaire and personal interviews during field trips.

The investigation revealed that, the traditional healers used 85 species of plants distributed in 76 genera belonging to 41 families to treat various diseases. The documented medicinal plants were mostly used to cure skin diseases, poison bites, stomachache and nervous disorders. In this study the most dominant family was Euphorbiaceae and leaves were most frequently used for the treatment of diseases.

This study showed that many people in the studied parts of Kancheepuram district still continue to depend on medicinal plants at least for the treatment of primary healthcare. The traditional healers are dwindling in number and there is a grave danger of traditional knowledge disappearing soon since the younger generation is not interested to carry on this tradition.

## Background

Plants have been used in traditional medicine for several thousand years [1]. The knowledge of medicinal plants has been accumulated in the course of many centuries based on different medicinal systems such as Ayurveda, Unani and Siddha. In India, it is reported that traditional healers use 2500 plant species and 100 species of plants serve as regular sources of medicine [2]. During the last few decades there has been an increasing interest in the study of medicinal plants and their traditional use in different parts of the world [3-7]. Documenting the indigenous knowledge through ethnobotanical studies is important for the conservation and utilization of biological resources.

Today according to the World Health Organization (WHO), as many as 80% of the world's people depend on traditional medicine for their primary healthcare needs. There are considerable economic benefits in the development of indigenous medicines and in the use of medicinal plants for the treatment of various diseases [8]. Due to less communication means, poverty, ignorance and unavailability of modern health facilities, most people especially rural people are still forced to practice traditional medicines for their common day ailments. Most of these people form the poorest link in the trade of medicinal plants [9]. A vast knowledge of how to use the plants against different illnesses may be expected to have accumulated in areas where the use of plants is still of great importance [10].

In the developed countries, 25 per cent of the medical drugs are based on plants and their derivatives [11]. A group of World Health Organization (WHO) experts, who met in Congo Brazzaville in 1976, sought to define traditional African medicine as 'the sum total of practices, measures, ingredients and procedures of all kinds whether material or not, which from time immemorial has enabled the African to guard against diseases, to alleviate his/her suffering and to cure him/herself' [12]. Traditional medical knowledge of medicinal plants and their use by indigenous cultures are not only useful for conservation of cultural traditions and biodiversity but also for community healthcare and drug development in the present and future [2].

Ethnobotany is not new to India because of its rich ethnic diversity. Jain [13] printed out that there are over 400 different tribal and other ethnic groups in India. The tribals constitute about 7.5 percent of India's population. During the last few decades there has been an increasing interest in the study of medicinal plants and their traditional use in different parts of India and there are many reports on the use of plants in traditional healing by either tribal people or indigenous communities of India [14-21]. Apart from the tribal groups, many other forest dwellers and rural people also posses' unique knowledge about plants [13].

The objective of this study was to interact with local traditional healers and document their knowledge on medicinal plants, their usage and the types of diseases treated etc. Kancheepuram is one of the 30 districts in Tamil Nadu and the traditional healing systems are still popular here. The present-day traditional healers are very old. Due to lack of interest among the younger generation as well as their tendency to migrate to cities for lucrative jobs, wealth of knowledge in this the area is declining. So far no systematic ethnobotanical survey has been made in this area and this is the first report on the medicinal plants used by the local traditional healers. A perusal of the literature reveals that, some of the ethnomedicinal works has been done in the forests of nearest districts in Tamil Nadu in the last two decades [22-25]. During the course of exploration of ethnomedicinal plants of the district, the informations have been gathered from the healers of rural villages found near forest areas where the people depend mostly on forests for their need and have sound knowledge of herbal remedies.

## Materials and methods

### The study area and ethnobotanical survey

Tamil Nadu is the 11^th ^largest state in India with a geographical area of 130058 km^2 ^and lies between 11° 00' to 12° 00' North latitudes and 77° 28' to 78° 50' East longitudes. The total forest cover Tamil Nadu is 21482 km^2 ^(16.52%). This includes 12,499 km^2 ^of dense forests (9.61%) and 8,963 km^2 ^of open forests (6.91%). Of the total forest area of Tamil Nadu, 3305 km^2 ^are under protected area (15%) which includes, 8 Wildlife sanctuaries, 12 Bird sanctuaries, 5 National parks, 3 Biosphere reserves and one Tiger reserve [26].

**Figure 1:**
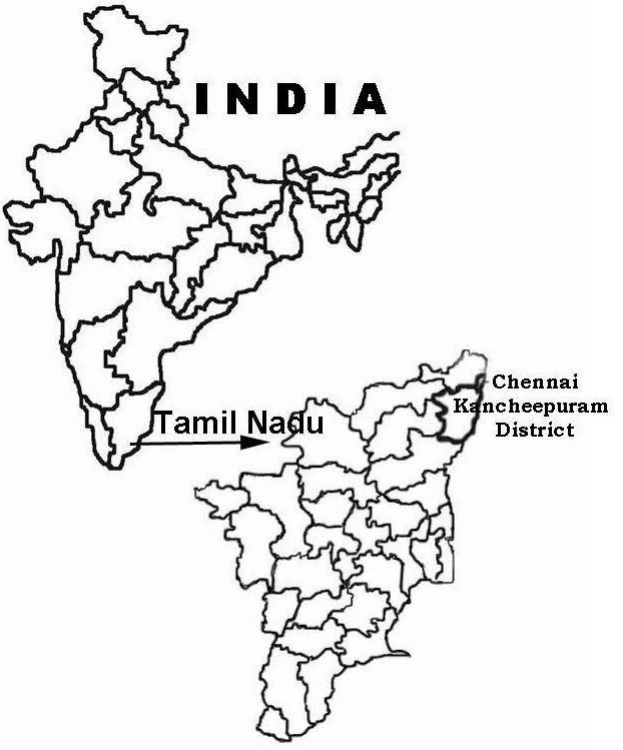
Location of the study area.

Kancheepuram district in north Tamil Nadu (Fig. 1) is placed 19^th ^in the forest cover with a geographical area of 4474 Sq.kms and recorded forest in the district is 5.32% [26]. This district is situated on the northern East Coast of Tamil Nadu and is adjacent to Bay of Bengal and Chennai city and is bounded in the west by Vellore and Thiruvannamalai districts, in the north by Thiruvallur and Chennai districts, in the south by Villupuram district and in the east by Bay of Bengal [27]. Paddy is the major crop cultivated in this district followed by groundnuts, sugarcane, cereals, millets and pulses. The river Palar is one of the most important rivers running through the district. There are only a few hills of considerable elevation in the district [27].

### Local traditional healers

Local traditional healers having practical knowledge of plants in medicine were interviewed in 12 villages of the district during October 2003 – April 2004. During the course of the study, four field trips were carried out in the study area totaling 40 days. Methods of selecting informants depended upon the distribution of local people having folk knowledge. They were requested to collect specimens of the plants they knew or to show the plant species on site. These informants were traditional healers themselves or had tradition of healing in their families and had knowledge of the medicinal use of the plants. Fuel wood from the surroundings was the main energy source for cooking and eating. The wealth of medicinal plant knowledge among the people of this district is based on hundreds of years of beliefs and observations. This knowledge has been transmitted orally from generation to generation; however it seems that it is vanishing from the modern society since younger people are not interested to carry on this tradition.

### Interview with traditional healers

Adopting the methods of Jain [28], ethnomedicinal data were collected through general conversations with the informants. The questionnaires were used to obtain information on medicinal plants with their local names, parts used, mode of preparation and administration. A total of 10 informants, comprising 8 males and 2 females were identified between the ages of 48 and 74. They were selected based on their knowledge of medicinal plants either for self-medication or for treating others. Informants were asked to come to field and show the plants with local name; the species mentioned by the informants were taxonomically identified.

### Preservation of plant specimens

Standard method was followed with regard to collection of plant materials, drying, mounting, preparation and preservation of plant specimens [29]. Voucher specimens of medicinal plants in triplicates were collected, prepared and identified. Plants with their correct nomenclature were arranged alphabetically by family name, vernacular name and ethnomedicinal uses. The identification and nomenclature of the listed plants were based on The Flora of Presidency of Madras [30] and The Flora of Tamil Nadu Carnatic [31]. They were later verified at Botanical Survey of India, Southern Circle, Coimbatore, India. All the preserved specimens were deposited at the Herbarium of Entomology Research Institute, Loyola College, Chennai.

## Results

The results of the survey are presented in Table 1 and the families of the plants are arranged in alphabetical order. The present investigation comprises 85 species of ethnomedicinal plants distributed in 76 genera belonging to 41 families. For each species botanical name, family, local name, parts used, methods of preparation, administration and ailments treated are provided. Traditional healers are using these plants to cure diseases related to skin problems, cold, fever, cough, headache, diarrhoea, fertility problems, toothache, stomach ache, wounds, diabetes, rheumatism, asthma, dysentery, small pox, bone fractures, earache, hair loss and poison (snake, scorpion and insect) bites (Figure 4).

**Table 1:** Medicinal plants used by traditional healers from Kancheepuram district of Tamil Nadu

**Botanical Name, Voucher Specimen Number and Family**	**Local Name (Tamil)**	**Method of preparation and Medicinal uses**
**Acanthaceae**		
*Adhatoda vasica *Nees (T464)	Adathodai	Leaves are ground with the flowers of *Hibiscus rosa-sinensis *and taken orally to treat asthma.
*Andrographis paniculata *(Burm. f.) Wallich ex Nees (T465)	Nilavembu	Leaf paste is applied topically at the bitten site of snake, beetle and scorpion. Powdered leaf is mixed with cow or goat's milk and taken orally to treat diabetes.
*Asystasia gangetica *(L.) T.Anderson (T224)	Valukkai keerai	Leaf powder is mixed with coconut oil and applied topically to heal wounds (burns).
**Aizoaceae**		
*Trianthema portulacastrum *L. (T466)	Saaranai	Decoction of roots is taken internally to treat Constipation and asthma.
**Amaranthaceae**		
*Achyranthes aspera L. *(T65)	Naayuruvi	Leaf paste is applied topically to treat cuts and Wounds.
*Aerva lanata *(L.) Juss.Ex Schult. (T412)	Siru peelai	Juice of whole plant is taken orally to treat cough, sore throat and wounds.
**Anacardiaceae**		
*Mangifera indica *L. (T472)	Maamaram	The latex from leaf and stem bark is used to treat heel cracks.
*Odina wodier *Roxb. Fl. (T475)	Uthiyam, Odimaram	Juice of leaves is taken orally to prevent white discharge in women.
**Annonaceae**		
*Polyalthia longifolia *(Sonn.) Thwaites. (T490)	Nettilingam	Juice extracted from the fresh stem bark is taken orally to treat indigestion.
**Apocynaceae**		
*Catharanthus roseus *G. Don. (T474)	Nithyakalyani	Whole plant is powdered and mixed with cow's milk and taken orally to treat diabetes.
*Nerium oleander *Sol. (T481)	Arali	Juice prepared from the stem bark is boiled with gingelly oil and two drops are poured into ear to treat ear pain.
*Rauwolfia tetraphylla *Linn. (T354)	Nagamani	Paste of the whole plant is mixed with castor oil and applied topically to treat skin diseases.
*Wrightia tinctoria *(Roxb.) R.Br. (T355)	Veppalai	Juice of seeds taken orally to treat indigestion.
**Araceae**		
*Acorus calamus *L. (T476)	Vasambu	Dried rhizome is ground in water and the paste is given orally to children for clarity of speech.
**Asclepiadaceae**		
*Gymnema sylvestre *R. Br. (T225)	Sirukurinchan	Leaf powder is mixed with cow's milk and taken orally to treat diabetes. The root powder is taken orally and also applied on the bitten spot to treat snake bite.
*Hemidesmus indicus *Linn. R. Br.Muell. (T237)	Nannari	Juice extracted from the whole plant is taken Internally to keep the body cool.
*Wattakaka volubilis *Cooke. (T298)	Kurinjan	Leaf paste is applied topically to treat rheumatic pain, cough, fever and severe cold.
**Asteraceae**		
*Eclipta prostrata *L. (T334)	Karisalanganni	Leaf powder is mixed with coconut oil & applied on the hair regularly for healthy and black hair.
*Sphaeranthus indicus *L. (T501)	Kottaikkarantai	Leaf, flower and seeds are ground into paste and applied topically to treat skin diseases and piles.
*Tridax procumbens *L. (T10)	Vettukayapundu	Leaf paste is applied topically on cuts and wounds
**Boraginaceae**		
*Coldenia procumbens *L. (T19)	Cheruppadai	Juice of leaf is taken orally to prevent white discharge in women.
*Heliotropium indicum *L. (T498)	Thelkodukku	Paste of whole plant is applied topically to treat wounds and skin affections.
**Caesalpiniaceae**		
*Cassia absus *L. (T477)	Karunaikanam	Seeds are ground into paste and applied topically to treat skin diseases and headache.
*Cassia auriculata *L. (T24)	Aavarai	Flowers are crushed and mixed with goat's milk and taken orally to prevent white discharge in women.
*Cassia occidentalis *L. (T497)	Peithagarai	Leaf paste is applied topically to treat scabies and to heal bone fractures.
*Tamarindus indica *L. (T100)	Puliya maram	Dried fruits are taken orally to treat eye infections.
**Capparaceae**		
*Capparis zeylanica *L. (T395)	Kathotti	Root bark is ground with water, boiled and taken orally to treat indigestion.
*Cleome viscosa *L. (T78)	Naikadugu,	Leaf paste is applied topically to heal wounds.
**Combretaceae**		
*Terminalia arjuna *Roxb.Ex. Dc Wight & Arn. (T478)	Marutha maram, Arjuna maram	Fruit paste is applied topically on wounds. Bark powder is boiled with water and inhaled to cure headache to kill worms in teeth.
**Convolvulaceae**		
*Merremia emarginata *(Burm.f.) Hall.f. (T479)	Elikkadilai	Decoction of the whole plant is taken internally to treat stomach problems.
**Cucurbitaceae**		
*Coccinia grandis *(L.) J. Voigt. (T226)	Kovai	Leaf Juice is mixed with butter and applied topically to treat skin diseases.
*Mukia maderaspatana *(L.) M. Roemer (T324)	Musumusukai	Leaf powder is mixed with boiled rice and taken orally to treat cold and cough.
**Cyperaceae**		
*Cyperus rotundus *L. (T480)	Korai, Muthakkasu	Paste of dried tuber is applied on breast of women to secrete more milk and applied topically on bitten site of scorpion.
**Euphorbiaceae**		
*Acalypha indica L. *(T496)	Kuppaimeni	Leaf paste is applied topically to treat skin diseases.
*Euphorbia antiquorum *Linn (T481)	Sathurakkalli	Dried latex is taken internally in low dose to help free motion.
*Euphorbia hirta *L. (T104)	Amman pacharisi	The milky latex is applied topically to treat wounds and lip cracks.
*Euphorbia tirucalli *L. (T495)	Kodikalli	The stem is boiled with water and given to children to treat skin diseases.
*Phyllanthus amarus *Schum. & Thnn. (T482)	Keezhanelli	Fresh leaves are ground and mixed with a cup of cow or goat's milk and taken internally to cure jaundice.
*Phyllanthus emblica *L. (T494)	Nelli	Fruit powder is mixed with cow's or goat's milk and taken orally to treat cold and cough.
*Ricinus communis *L. (T502)	Amanakku	The leaf juice is taken orally or washed leaves are tied on the breast to increase secretion of milk in women. The oil prepared from the seeds is applied on lower stomach to get relief from stomachache.
**Fabaceae**		
*Abrus precatorius *Linn. (T03)	Kundumani	Root powder is taken orally along with cow's milk to treat scorpion sting and snakebite.
*Clitoria ternatea *L. (T206)	Sangu Pushpam	Root powder is mixed with water and taken orally to treat indigestion, eye diseases and headache.
*Pongamia pinnata *(L.) Pierre.(T503)	Punga maram	Juice of root is mixed with equal amount of coconut milk, boiled and applied topically to cure wound and gastric trouble.
**Lamiaceae**		
*Coleus aromaticus *Benth. (T483)	Karpuravalli	Leaf juice is taken orally by children to treat Indigestion and cough.
*Leucas aspera *(Willd.) Link. (T66)	Thumbai	A bunch of leaves is boiled and the vapour is inhaled to cure head ache and fever.
*Ocimum sanctum *L. (T504)	Thulasi, Tulsi	Leaves are crushed with onion bulbs and the juice is taken orally to treat cough, cold and headache.
**Lauraceae**		
*Cinnamomum verum *Presl. (T493)	Lavangappattai, Karuvappatttai	Decoction of stem bark is taken internally to treat cough, dysentery and to keep the body cool.
**Liliaceae**		
*Aloe vera *L. (T484)	Sothukathalai	Sap mixed with oil is heated and the mixture is applied on hair for hair growth and good sleep.
*Sanservieria roxburghiana *Schult. (T505)	Marul	Juice of warmed leaf is poured into ear to treat ear pain.
**Lythraceae**		
*Lawsonia inermis *L. (T492)	Maruthani	Leaf powder is mixed with coconut oil and applied topically to treat cuts and wounds.
**Malvaceae**		
*Abutilon indicum *L. (T485)	Thuthi	Leaf juice and root are taken orally to treat dental problems.
*Hibiscus rosa-sinensis *L. (T506)	Semparuthi	Paste of fresh leaves is applied on the hair for healthy and black hair.
*Sida acuta *Burn. (T491)	Arival manai poondu	Leaf paste is applied topically to heal cuts, wounds and to get relief from headache.
**Meliaceae**		
*Azadirachta indica *A. Juss. (T552)	Vembu	Leaf paste is applied topically on the body to treat small pox, rheumatism and skin diseases. The young twigs are used as toothbrush to develop strong teeth.
**Menispermaceae**		
*Tinospora cordifolia *Miers. (T523)	Seendil	Leaf paste is applied topically to treat wounds.
**Mimosaceae**		
*Acacia leucophloea *(Roxb.) Willd. (T490)	Velvelamaram	Paste of fresh stem bark is applied topically to treat cuts and wounds.
*Mimosa pudica L*.(T238)	Thottasurungi	Pinch of leaf paste is applied topically to treat cuts and wounds.
**Moraceae**		
*Ficus benghalensis *L. (T551)	Alamaram	Stem latex is applied topically on heel cracks. Young stem is used as tooth brush.
*Ficus racemosa *L. (T 486)	Athi maram	Stem latex is applied topically to treat heel cracks.
*Ficus religiosa *L. (T550)	Arasu	Dried leaf powder is mixed with water and takenorally to get relief from body pain.
**Moringaceae**		
*Moringa oleifera *Lam. (T511)	Murangai	The leaf is taken as food and it reduces body heat and to treat indigestion and eye diseases. Flower is taken as food and it gives chillness to Eyes and increases sperm production in men.
**Myrtaceae**		
*Syzygium cumini *(L.) Skeels (T489)	Naval maram	Paste of stem bark is applied topically to treat swellings. The ripe fresh fruits are taken orally to reduce body heat.
**Nyctaginaceae**		
*Boerhaavia diffusa *L. (T522)	Mookaratai	Root paste is applied topically to treat Hydrocele.
**Poaceae**		
*Cynodon dactylon *L. Pers. (T487)	Arugampullu	Decoction of whole plant is taken orally to keep the body cool.
**Rhamnaceae**		
*Zizyphus mauritiana *Lam.(T401)	Ilandai	Leaf and bark decoction is boiled and it is used to take bath to treat severe body pain. Dried bark powder is applied topically to treat wounds.
**Rubiaceae**		
*Morinda tinctoria *Roxb. (T511)	Nuna, Manjanathi	Leaf juice is given orally to children before food for easy digestion.
*Oldenlandia umbellata *L. (T585)	Siruver, Sayaver	The root paste is applied topically to arrest bleeding.
*Spermacoce hispida *L (T591)	Nathaichuri	The seeds are crushed into paste and taken orally to treat stomach problems.
**Rutaceae**		
*Aegle marmelos *Corr.ex.Roxb (T561)	Vilvam	Leaf paste is applied topically to heal wounds.
*Citrus aurantifolia *(Christm.) Swingle. L. (T534)	Elumitchai	Decoction of leaves is inhaled to get relief from fever, headache and cold.
*Murraya koenigii (*L.) Sprengel (T561)	Karuveppilai; Kari-vembu	Juice of tender leaves is taken orally to arrest vomiting.
**Sapindaceae**		
*Cardiospermum halicacabum *L. (T549)	Mudakkathan	Root is boiled with oil and applied on head before bath to treat throat infection and headache.
**Solanaceae**		
*Datura metel *L.(T535)	Oomathai	Few drops of leaf juice is poured into ear to treat earache.
*Solanum nigrum *L. (T526)	Manathakkali	Whole plant parts are taken as food to treat cough.
*Solanum torvum *Sw. (T527)	Sundaikkai	Leaf juice is taken orally to reduce body heat.
*Solanum trilobatum *L. (T558)	Thuthuvalai	Unripe fruits are prepared as curry or roasted in gingelly oil and taken orally along with food to strengthen the body. The leaf juice is taken orally to treat cough and itching.
**Sterculiaceae**		
*Melochia corchorifolia *L. (T110)	Punnakku chedi	Boiled leaf is taken as food to help in free motion.
**Verbenaceae**		
*Clerodendrum inerme (*L.) Gaertn (T181)	Piei nari sangu	Leaf is ground in water and the juice is taken orally to treat fever.
*Lantana camara *L. (T14)	Unni chedi	A handful of flower is ground with coconut oil and applied topically on the head to get relief from headache.
*Lippia nodiflora *Mich. (T560)	Poduthalai	Paste of leaves is applied topically to treat swellings and wounds.
*Stachytarpheta jamaicensis *Vahl. (T515)	Seemainayuruvi	Paste of stem and root bark is applied topically to treat dysentery.
*Vitex negundo *L. (T372)	Notchi	Leaves are boiled in water and the vapour is inhaled twice a day to get relief from headache, fever, cold, and cough.
**Violaceae**		
*Hybanthus enneaspermus *F. Muell (T520)	Orithal thamarai	Paste of whole plant is applied topically to treat cough.
**Vitaceae**		
*Cissus quadrangularis *L. (T444)	Pirandai	Paste of stem is taken orally for easy digestion.
**Zygophyllaceae**		
*Tribulus terrestris *L. (T567)	Nerunchimul	The fruit and root are mixed with boiled raw rice, taken orally to prevent white discharge in women and to treat urinary troubles.

**Figure 2:**
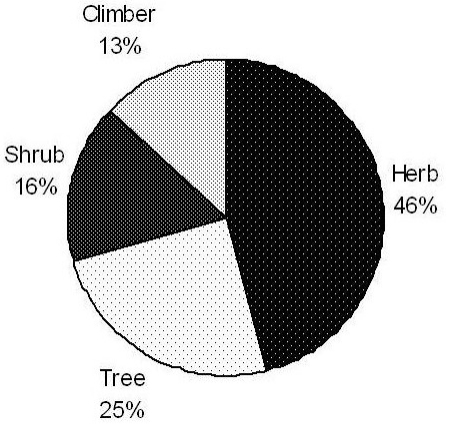
Habit.

Herbs (39 species) were found to be the most used plants (Figure 2) followed by trees (21 species), shrubs (14 species) and climbers (11 species) in descending order. The most dominant families in the study were Euphorbiaceae (7 species), Verbenaceae (5 species), Caesalpiniaceae, Solanaceae and Apocynaceae (4 species), Acanthaceae, Asclepiadaceae, Asteraceae, Fabaceae, Lamiaceae, Malvaceae, Moraceae, Rubiaceae and Rutaceae (3 species). Other families with low number are listed below: Amaranthaceae, Anacardiaceae, Boraginaceae, Capparaceae, Liliaceae and Cucurbitaceae (2 species), Aizoaceae, Annonaceae, Araceae, Combretaceae, Convolvulaceae, Cyperaceae, Lauraceae, Lythraceae, Meliaceae, Menispermaceae, Sapindaceae, Rhamnaceae, Poaceae, Nyctaginaceae, Myrtaceae, Moringaceae, Mimosaceae, Sterculiaceae, Violaceae, Vitaceae and Zygophyllaceae (1 species).

Local traditional healers are commonly using the following plants to treat more number of diseases. They are: *Vitex negundo*, *Wedelia calendulacea*, *Ocimum gratissimum*, *Cynodon dactylon*, *Solanum nigrum*, *Azadirachta indica*, *Pongamia pinnata*, *Aristolochia indica*, *Solanum trilobatum*, *Adhatoda vasica*, *Phyllanthus emblica*, *Ocimum sanctum*, *Murraya koenigii*, *Ficus benghalensis *and *Cassia auriculata*. Preference for their use may be related to their availability.

## Discussion

**Figure 3:**
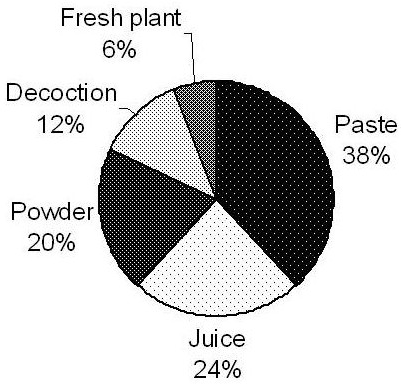
Method of preparation.

Different parts of medicinal plants were used as medicine by the local traditional healers. Among the different plant parts, the leaves were most frequently used for the treatment of diseases followed by whole plant parts, fruit, stem, root, stem and root bark, seed, flower and latex. The methods of preparation (Figure 3) fall into four categories, viz.: plant parts applied as a paste (38%), juice extracted from the fresh plant parts (24%), powder made from fresh or dried plant parts (20%), some fresh plant parts (6%), and decoction (12%). External applications (mostly for skin diseases, snake bites and wounds) and internal consumption of the preparations were involved in the treatment of diseases.

**Figure 4:**
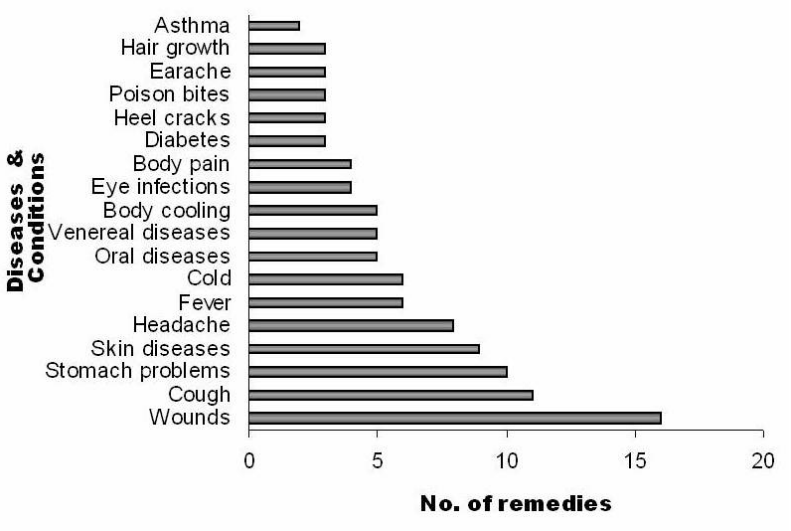
Number of remedies used for various ailments.

It was observed that, most of the remedies consisted of single plant part and more than one method of preparation. However, many of the remedies consisted of different parts of the same plant species to treat single or more diseases. For example, *Andrographis paniculata *– leaf paste is used to treat poison bites and leaf powder is used to treat diabetes; *Gymnema sylvestre *– root powder is used to treat poison bites and leaf powder is used to treat diabetes; *Ricinus communis *-juice extracted from leaves is used to increase secretion of milk and oil obtained from seeds is used to treat stomach ache; *Azadirachta indica *– leaf paste is used to treat small pox, rheumatism and skin diseases and young twigs are used as tooth brush; *Ficus benghalensis *– stem latex is used to treat heel cracks and young twigs are used as tooth brush; *Moringa oleifera *– boiled leaves are used to reduce body heat, to treat indigestion and eye diseases and flowers are used to cool the eyes and increase sperm production; *Zizyphus mauritiana *– decoction of leaf is used to get relief from body pain and bark powder is used to treat wounds; *Solanum torvum *– juice extracted from the leaf is used to reduce body heat and unripe fruits are used to strengthen the body.

Moreover a single plant is used for more than one disease. For example, *Andrographis paniculata *(diabetes and scorpion bites), *Trianthema portulacastrum *(constipation and asthma), *Aerva lanata *(cough, sore throat and wounds), *Gymnema sylvestris *(diabetes and poison bites), *Wattakaka volubilis *(rheumatic pain, cough, fever and severe cold), *Sphaeranthus indicus *(skin diseases and piles), *Heliotropium indicum *(wounds and skin affections), *Cassia absus *(wounds and skin affections), *Cassia occidentalis *(scabies and bone fractures), *Terminalia arjuna *(wounds, headache and tooth infections), *Mukia maderaspatana *(cold and cough), *Cyperus rotundus *(milk secretion and scorpion bites), *Euphorbia hirta *(wounds and lip cracks), *Phyllanthus emblica *(cold and cough), *Ricinus communis *(milk secretion ad stomachache), *Clitoria ternatea *(eye diseases and headache), *Pongamia pinnata *(wound and gas trouble), *Coleus aromaticus *(indigestion and cough),*Leucas aspera *(headache and fever), *Ocimum sanctum *(cough, cold and headache), *Cinnamomum verum *(cough, dysentery and body cooling), *Aloe vera *(hair loss and good sleep), *Azadirachta indica *(small pox, rheumatism and skin diseases), *Moringa oleifera *(body heat, indigestion, eye diseases and to increases sperm production), *Syzygium cumini *(swellings and body heat), *Zizyphus mauritiana *(body pain and wounds), *Citrus aurantifolia *(fever, headache and cold), *Cardiospermum halicacabum *(throat infection and headache), *Solanum torvum *(to reduce body heat and to strengthening the body), *Solanum trilobatum *(cough and itching) and *vitex negundo *(headache, fever, cold and cough) and these recipes are prepared using different ingredients of non-plant origin such as water, salt, honey, etc.

Common health problems in the sites of the study area were skin problems such as wounds, cuts, burns and skin diseases and the largest number of the remedies (wounds – 16 remedies and skin diseases – 9 remedies) was used to treat these troubles. Several studies have enumerated the plants used for wound healing and skin diseases in various parts of the world [14,32-34]. 24 remedies were used to alleviate problems of the respiratory system such as cold, cough and asthma. Most of the plants in Lamiaceae were used to treat cold, cough, fever, headache and asthma. Ghorbani [35] reported 16 plant species that were used for respiratory diseases in north Iran and safety and efficacy of the treatments for respiratory track infections were reviewed [36]. Common ailments such as headaches or coughs are considered to be diseases with natural causes and hence their symptoms are treated at the household level, without resource to magical practices [12]. In the present study eight remedies (*Terminalia arjuna, Leucas aspera, Ocimum sanctum, Sida acuta, Citrus aurantifolia, Cardiospermum halicacabum, Lantana camara *and *Vitex negundo*) were used to get relief from headache. Recently Ignacimuthu et al [37] reported that *Ceropegia candelabrum*, *Pergularia daemia *and *Vitex negundo *were used by tribals for the treatment of headache.

Traditional healers of Kancheepuram district used nine plant species to treat stomach problems (three plants to treat stomachache and six plants to cure digestive problems). In North-Western Patagonia the people of Curruhuinca community were affected with digestive problems and the highest fidelity level was found for species utilized for treating digestive ailments [38] and Ghorbani [35] reported that there were 48 plants for the treatment of gastrointestinal disorders in north of Iran. The tribal people of Western Madhya Pradesh of India used 13 plants for the treatment of jaundice [19]. In the present study only *Phyllanthus amarus *was used for the treatment of jaundice. *Abutilon indicum, Azadirachta indica, Ficus benghalensis *and *Terminalia arjuna *were used to treat dental problems. Various studies have reported on the indigenous use of medicinal plants in the treatment of oral diseases [[39] &[21]]. *Andrographis paniculata, Catharanthus roseus *and *Gymnema sylvestre *were used to treat diabetes by the local traditional healers. Chherti et al [15] reported that the tribal people of Sikkim and Darjeeling Himalayan region in India utilized 37 species of plants belonging to 28 different families as antidiabetic agents.

## Conclusion

The survey indicated that, the study area has plenty of medicinal plants to treat a wide spectrum of human ailments. Earlier studies on traditional medicinal plants also revealed that the economically backward local and tribal people of Tamil Nadu prefer folk medicine due to low cost and sometimes it is a part of their social life and culture [40-45]. It is evident from the interviews conducted in different villages, knowledge of medicinal plants is limited to traditional healers, herbalists and elderly persons who are living in rural areas. This study also points out that certain species of medicinal plants are being exploited by the local residents who are unaware of the importance of medicinal plants in the ecosystem.

This study concluded that even though the accessibility of Western medicine for simple and complicated diseases is available, many people in the studied parts of Kancheepuram district is still continue to depend on medicinal plants, at least for the treatment of some simple diseases such as, cold, cough, fever, headache, poison bites, skin diseases and tooth infections. Well-knowledged healers have good interactions with patients and this would improve the quality of healthcare delivery. The present-day traditional healers are very old. Due to lack of interest among the younger generation as well as their tendency to migrate to cities for lucrative jobs, there is a possibility of losing this wealth of knowledge in the near future. It thus becomes necessary to acquire and preserve this traditional system of medicine by proper documentation and identification of specimens.
